# The Flavonoid Extract of *Polygonum viviparum* L. Alleviates Dextran Sulfate Sodium-Induced Ulcerative Colitis by Regulating Intestinal Flora Homeostasis and Uric Acid Levels Through Inhibition of PI3K/AKT/NF-κB/IL-17 Signaling Pathway

**DOI:** 10.3390/antiox14101206

**Published:** 2025-10-05

**Authors:** Haoyu Liu, Zhen Yang, Qian Chen, Hongjuan Zhang, Yu Liu, Di Wu, Dan Shao, Shengyi Wang, Baocheng Hao

**Affiliations:** Key Laboratory of New Animal Drug Project, Gansu Province, Key Laboratory of Veterinary Pharmaceutical Development, Ministry of Agriculture and Rural Affairs, Lanzhou Institute of Husbandry and Pharmaceutical Sciences of Chinese Academy of Agriculture Sciences, Lanzhou 730050, China; lhy000503@163.com (H.L.); yangzhen01@caas.cn (Z.Y.); 15528168849@163.com (Q.C.); zhanghongjuan@caas.cn (H.Z.); liuyu@caas.cn (Y.L.); wudi@caas.cn (D.W.); shaodan@caas.cn (D.S.)

**Keywords:** flavonoid extract of *Polygonum viviparum* L., ulcerative colitis, inflammation, intestinal barrier damage, metabolomics

## Abstract

Chronic inflammatory bowel disease, ulcerative colitis (UC), currently lacks specific drugs for clinical treatment, and screening effective therapeutic agents from natural plants represents a critical research strategy. This study aimed to investigate the therapeutic potential of the flavonoid extract of *Polygonum viviparum* L. (TFPV) against UC. Liquid chromatography-mass spectrometry (LC-MS) was used to identify the chemical components of TFPV, while cell and animal models were employed to evaluate its anti-inflammatory effects on lipopolysaccharide (LPS)-induced inflammation. The mechanism of anti-inflammatory action was further investigated using a mouse model of UC induced by dextran sulfate sodium (DSS). The results revealed the identification of 32 bioactive components in TFPV, with major compounds such as kaempferol, luteolin, galangin, and quercetin. TFPV effectively mitigated inflammatory damage induced by LPS in IPEC-J2 cells and C57BL/6 mice. In the UC modeled by DSS, TFPV attenuated intestinal inflammation by reducing pro-inflammatory cytokines IL-1β, IL-6, and TNF-α; increasing the anti-inflammatory cytokine IL-10; up-regulating tight junction protein expression such as Claudin-1, Occludin, and ZO-1; and inhibiting the expression of PI3K, AKT, NF-κB, and IL-17 proteins. Analysis of mice fecal samples through 16S rRNA gene sequencing demonstrated that TFPV adjusted the equilibrium of gut microbiota by boosting the abundance of *Dubosiella* and diminishing that of *Enterococcus*, *Romboutsia*, and *Enterobacter*. Untargeted metabolomics analysis further revealed that TFPV reduced inosine and ADP levels while increasing dGMP levels by the regulation of purine metabolism, ultimately resulting in decreased uric acid levels and thereby alleviating intestinal inflammation. Additionally, TFPV safeguarded the intestinal mucosal barrier by enhancing the expression of tight junctions. In conclusion, TFPV alleviates UC by blocking the PI3K/AKT/NF-κB and IL-17 signaling pathways, lessening intestinal inflammation and injury, safeguarding intestinal barrier integrity, balancing gut microbiota, and lowering uric acid levels, suggesting its promise as a therapeutic agent for UC.

## 1. Introduction

Ulcerative colitis (UC) is a chronic relapsing inflammatory bowel disease (IBD) [1] characterized by persistent mucosal inflammation and disruption of the epithelial barrier. It imposes a significant global health burden due to its increasing incidence and limited treatment efficacy [2,3]. Various theories exist regarding the pathogenesis of UC, such as heightened levels of intestinal inflammation, damage to the intestinal barrier, and alterations in the gut microbiota [4]. Aberrant intestinal inflammation is closely related to signaling pathways like NF-κB and IL-17 [5,6]. Abnormal activation of the NF-κB pathway is a pivotal driver of intestinal inflammation, regulating pro-inflammatory cytokines, affecting the intestinal barrier, and modulating immune cell function. IL-17, secreted by Th17 cells, triggers downstream pathways through receptors to amplify the inflammatory response. Complications of UC can include anemia, intestinal perforation, and cancer [7]. Current medications, including aminosalicylates, corticosteroids, immunomodulators, and monoclonal antibodies, primarily target achieving sustained symptom relief. However, long-term use of these drugs may cause immunosuppression, with symptom control remaining the primary focus of UC treatment [8].

Emerging evidence emphasizes the crucial role of the gut microbiota–metabolite axis in modulating microbial ecology and host immune homeostasis during the progression of IBD [9,10,11,12]. The indigenous gut microbiota serves as a physiological barrier, safeguarding the host intestine against pathogenic microorganisms [13]. Alterations in the gut microbiota composition disturb the symbiotic relationship between the microbial community and the host. In most healthy individuals, Firmicutes, Bacteroidetes, Proteobacteria, and Actinobacteria collectively constitute around 99% of the gut microbiota, with Firmicutes and Bacteroidetes comprising approximately 90% of the total microbiota [14].

In patients with UC, there is a notable decline in microbial diversity, characterized by a diminished Firmicutes/Bacteroidetes ratio and anomalous metabolite profiles such as sphingolipids and cholesterol [15]. This dysregulation not only initiates immune and barrier dysfunction but also correlates closely with disturbances in purine metabolism. Research indicates that the imbalance in gut microbiota can impact purine metabolism through various pathways, thereby significantly influencing the progression of UC. Fusobacterium nucleatum, a pathogen associated with periodontitis, exhibits abnormal colonization in the intestines of UC patients, leading to heightened intestinal inflammation, epithelial barrier dysfunction, microbiota dysregulation, and metabolic irregularities, particularly exacerbating abnormalities in uric acid metabolism [16]. As the final product of purine metabolism, uric acid levels directly reflect the purine metabolic status of organisms. Clinical studies have demonstrated common alterations in uric acid metabolism among patients with IBD, particularly in those with UC, who exhibit a notable association with gout (OR 1.38, CI: 1.31–1.44). This association is even more pronounced in UC patients who have undergone intestinal resection, underscoring the inherent connection between UC, dysregulated gut microbiota, and abnormalities in purine metabolism [17]. Recent research has shed light on potential therapeutic avenues for UC. Administering healthy mice with Gm-EVsCN@Lp127-trained bacteria orally not only alleviated colitis, facilitated mucosal healing, and regulated the gut microbiota but also modulated purine metabolism, leading to a significant reduction in uric acid levels and a consequent improvement in UC symptoms [18]. These findings suggest that targeting purine metabolism through gut microbiota modulation could represent a potential strategy for treating UC. Metabolites generated by the gut microbiota serve as pivotal mediators linking the microbiota and the host [19,20]. This triad of microbiota–metabolite–immunity underscores the importance of therapies that address both inflammatory pathways and the restoration of the microbial ecosystem, with a specific focus on the regulation of purine metabolism.

Currently, there remains a dearth of specific pharmaceutical interventions for UC in clinical practice. Therefore, there is a pressing need to investigate therapeutic agents with multi-target effects sourced from natural foods. *Polygonum viviparum* L. (PV), an indigenous Chinese medicinal plant, exhibits diverse pharmacological activities, such as anti-inflammatory and antioxidant. Its bioactive components include flavonoids, tannins, and saponins [21]. Traditionally, PV has been utilized for ailments such as dysentery, enteritis, and sore throat. Notably, it serves as the primary food for the indigenous insects of the rare medicinal herb *Ophiocordyceps sinensis* [22]. While previous studies have provided initial insights into the phytochemical composition of TFPV, its potential application in UC therapy remains underexplored. This study aims to characterize the chemical composition of TFPV using LC-MS and investigate its effects on intestinal inflammation, intestinal barrier function, intestinal flora, and endogenous metabolites in DSS-induced UC mice. These findings offer a foundation for the clinical application of TFPV in UC treatment and future drug development endeavors.

## 2. Materials and Methods

### 2.1. Chemicals and Reagents

*Polygonum viviparum* L. (PV) was purchased from Gansu Fuxinghou Biomedical Technology Co., Ltd. (Lanzhou, China). The porcine intestinal epithelial cell line (IPEC-J2) was provided by the Cell Culture Center of the Chinese Academy of Sciences (Shanghai, China). Lipopolysaccharide (LPS) derived from *E*. *coli* 055: B5 was purchased from Sigma-Aldrich (St. Louis, MO, USA). Dextran sulfate sodium (DSS) was purchased from MP Biomedicals (Santa Ana, CA, USA). Salicylazosulfapyridine (SASP) was obtained from Shanghai Xinyi Tianping Pharmaceutical Co., Ltd. (Shanghai, China). Dexamethasone (DEX) was sourced from Chenxin Pharmaceutical Co., Ltd. (Jining, China). Enzyme-linked immunosorbent assay (ELISA) kits of mouse TNF-α, IL-1β, IL-6, IL-10, and BCA protein concentration determination kits were purchased from Beijing Solarbio Science & Technology Co., Ltd. (Beijing, China). Antibodies of Claudin-1, Occludin, ZO-1, NF-κB, IL-17, and β-actin were bought from Abcam Technology Inc. (Boston, MA, USA). Enhanced chemiluminescence (ECL) and the molecular weight standard of the predicted protein are sourced from Ncmbio Co., Ltd. (Suzhou, China). PrimeScript™ RT reagent kit with gDNA eraser and TB Green^®^ Premix Ex Taq^TM^ II (Tli RNaseH Plus) were bought from Takara Bio (Shiga, Japan). The qPCR primers were obtained from Sangon Biotech (Shanghai, China). Acetylsalicylic acid (aspirin) was bought from Aladdin Bio-Chem Technology Co., Ltd. (Shanghai, China). Fluorescein isothiocyanate (FITC) conjugated goat anti-rabbit IgG (H + L) and Cyanine-3 (Cy3) conjugated goat anti-rabbit IgG (H + L) were purchased from Servicebio Technology Co. Ltd. (Wuhan, China). Cell culture materials were obtained from Life Technologies (Gibco, Grand Island, NY, USA). Analytical grade formic acid, chromatographic grade methanol, acetonitrile, and isopropanol were purchased from Thermo Fisher Scientific (Waltham, MA, USA).

### 2.2. Preparation and Identification of TFPV

TFPV was extracted with 70% ethanol at a 1:10 solid–liquid ratio, repeated three times, then purified by AB-8 microporous adsorption resin, then concentrated and freeze-dried. The components were analyzed by HPLC-QTOF/MS. The chromatographic column was C18 (250 × 4.6 mm, 5 mm). The mobile phase was acetonitrile (solvent A) and 0.1% (*v*/*v*) formic acid (solvent B), and the gradient elution procedure was 0–3 min, 5–5% A; 3–23 min, 5–95% A; 23–28 min, 95–95% A; 28–30 min, 95–5% A, flow rate 0.4 mL/min. The temperature of the column box was 35 °C, the sample size was 10 μL, and the wavelength was 360 nm.

### 2.3. Cell Culture

IPEC-J2 cells were cultured in DMEM/F12 medium (Gibco) supplemented with 10% fetal bovine serum (FBS), 100 IU/mL penicillin, and 100 µg/mL streptomycin at 37 °C in a fully humidified incubator containing 5% CO_2_ [23]. When grown as a dense monolayer, the cells were routinely subcultured to the next generation.

### 2.4. Cell Viability Assay and Establishment of LPS-Induced Inflammatory Model in IPEC-J2 Cells

The effects of TFPV on the cell viability of IPEC-J2 cells were analyzed using a previously described procedure with minor modifications [24]. IPEC-J2 cells were seeded in 96-wells plates at a density of 1 × 10^4^ per well and cultured for 4 h. Subsequently, the blank control group was treated with culture medium for 30 h; the LPS group was treated with culture medium for 6 h at first, followed by adding LPS (10 µg/mL) for another 24 h stimulation; the TFPV groups were pretreated with different concentrations of TFPV, respectively (1, 2.5, 5, 10, 20, and 40 µg/mL) for 6 h and then stimulated with LPS (10 µg/mL) for 24 h. After incubation, the original medium was discarded, and 100 µL of 10% CCK-8 solution was added to each well. The cell viability in each group was measured at 450 nm after 4 h of CCK-8 addition by using an enzyme-linked immunosorbent assay (ELISA) detector (Gene Company Limited), and the cell viability was expressed as the percentage viability according to the following formula:

Cell Viability (%) = [(absorbance of treatment − absorbance of blank)/(absorbance of control − absorbance of blank)] × 100%

### 2.5. Animals

C57BL/6 mice (SPF grade, male, weight: 18–22 g) were obtained from the Experimental Animal Center of Lanzhou Veterinary Research Institute of Chinese Academy of Agricultural Sciences (Lanzhou, China). A total of 88 mice were used in the study, among which 48 were employed for the LPS-induced anti-inflammatory experiment and 40 for the DSS-induced ulcerative colitis experiment. All mice were housed in an SPF facility (20–24 °C, relative humidity: 40–60%, 12 h light–dark cycle) in the Lanzhou Institute of Animal Husbandry and Pharmaceutical Sciences of the Chinese Academy of Agricultural Sciences. Standard mouse food and drinking water were provided adlibitum. All procedures involving animals were performed according to the Guidelines for the Care and Use of Laboratory Animals published by the United States National Institute of Health (NIH, Publication No. 85-23, 1996) and approved by the Experimental Animal Ethics Committee of the Lanzhou Institute of Animal Husbandry and Pharmaceutical Sciences of Chinese Academy of Agricultural Sciences (Ethics Licenses No. 2023-025 and No. 2023-026).

### 2.6. Establishment of LPS-Induced Intestinal Inflammation Model in Mice

After one week of acclimatization, mice were randomly divided into six groups, with eight mice in each group, namely the control group (CON), lipopolysaccharide group (LPS), positive drug group (DEX), low-dose group of TFPV (L), medium-dose group of TFPV (M), and high-dose group of TFPV (H). The DEX group was given dexamethasone (1 mg/kg·bw) by gavage per day, while the L, M, and H groups were given TFPV (5, 10, and 20 mg/kg·bw, respectively) by gavage per day. After 7 consecutive days of administration, mice in the control group were intraperitoneally injected with 0.2 mL of normal saline, and mice in the other groups were intraperitoneally injected with LPS (10 mg/kg·bw, 0.2 mL) to establish the intestinal inflammation model. The control group mice were intraperitoneally injected with 0.2 mL of physiological saline. After 18 h of injection, all mice were weighed, anesthetized, executed, and dissected, and organ tissues and serum were collected [25].

### 2.7. Network Pharmacology Analysis

Network pharmacology analysis was conducted via an off-target analysis approach. Initially, the active components were meticulously screened using the Traditional Chinese Medicine Systems Pharmacology (TCMSP) database (https://www.tcmsp-e.com/) (accessed on 20 September 2024). Components were selected based on strict criteria: those characterized by high gastrointestinal absorption as described in the Swiss ADME database and with at least two drug-like properties meeting the “yes” standard. Subsequently, the SMILE structures of the selected components were retrieved from the PubChem database (https://pubchem.ncbi.nlm.nih.gov/) (accessed on 20 September 2024), while their corresponding action targets were obtained from the SwissTarget Prediction database (http://www.swisstargetprediction.ch/) (accessed on 20 September 2024).

For disease-related targets, the Genecards database (https://www.genecards.org/) (accessed on 22 September 2024) was queried using “ulcerative colitis” as the search keyword. To visualize the Venn diagram representing the intersection of targets between the selected components and the disease, the online tool (http://jvenn.toulouse.inra.fr/app/example.html) (accessed on 22 September 2024) was employed. Protein–protein interaction (PPI) data were retrieved from the String database (https://cn.string-db.org/) (accessed on 22 September 2024) and then analyzed and visualized through the Cytoscape application (version 3.7.2).

Furthermore, Gene Ontology (GO) enrichment analysis encompassing biological processes (BP), cellular components (CC), and molecular functions (MF), as well as Kyoto Encyclopedia of Genes and Genomes (KEGG) pathway enrichment analysis of the screened targets (https://www.genome.jp/kegg/) (accessed on 24 September 2024), was performed using the DAVID database (https://david.ncifcrf.gov) (accessed on 25 September 2024). The analysis was specifically configured for “Homo sapiens”, and only results with a *p*-value less than 0.05 were considered statistically significant. Finally, molecular docking was executed using Pymol 2.4, with the core genes identified by network pharmacology and the components of TFPV serving as the docking targets.

### 2.8. Establishment of Ulcerative Colitis Model in Mice

Acute ulcerative colitis was induced in all three groups of mice except the control group (C) by administration of 3% DSS by gavage for one week [26]. Among them, the positive control group (P) and the TFPV group were treated with SASP and TFPV, respectively, at the same time of DSS stimulation. During the 3 days after modeling, the blank control group and DSS group (M) were left untreated except for free diet and water, while the positive control group and TFPV group continued to be given SASP and TFPV treatment, respectively. Calculate the Disease Activity Index (DAI) of mice daily and score them according to the reference literature [27,28]. At the end of drug administration, the mice were anesthetically executed and dissected, and the colons were collected and measured for length. The colon tissues were washed with PBS and then rapidly frozen in liquid nitrogen and stored at −80 °C for use.

### 2.9. Histopathological Evaluation

Colon tissues collected were fixed in 4% paraformaldehyde and embedded in paraffin. Then the colon were cut into sections and stained with hematoxylin and eosin (H&E). Images were observed by a microscope (Nikon Eclipse E100, Sendai, Japan) and captured using Nikon DS-U3 software (NIS-Elements).

### 2.10. Enzyme-Linked Immunosorbent (ELISA) Assay

The blood was collected and then centrifuged at 4 °C at 2000× *g* for 20 min. The supernatant was collected and stored at −80 °C. The TNF-α, IL-1β, IL-6, and IL-10 levels in supernatant were determined using ELISA kits according to the manufacturer’s recommendation. The ELISA kits for mouse TNF-α, IL-6, IL-1β, and IL-10 were provided by Solarbio Biotechnology Co., Ltd. (Beijing, China).

### 2.11. Immunohistochemical (IHC) Analysis

The expressions of Claudin-1, Occludin, and ZO-1 in the colon were detected by IHC analysis. Sections of colon tissue were blocked with 3% bovine serum albumin (BSA) for 30 min after incubation with antigen repair solution and then incubated overnight at 4 °C with primary antibodies recognizing Claudin-1, Occludin, and ZO-1 at the indicated concentrations. Subsequently, sections were washed three times with PBS and placed in secondary antibody coupled with horseradish peroxidase (HRP) and incubated for 50 min away from light. Photographs were taken using a microscope (Nikon Eclipse C1) and imaging Nikon DS-U3 software; observation and evaluation were performed using CaseViewer 2.4 (3DHISTECH, Budapest, Hungary).

### 2.12. RNA Extraction and Quantitative Real-Time PCR Analysis (RT-qPCR)

Total RNA of IPEC-J2 cells in each group was extracted using the Simply P Total RNA Extraction Kit (Bioflux, Hangzhou, China) and then reverse-transcribed into cDNA using the PrimeScript™ RT reagent Kit with gDNA Eraser (Takara, Shiga, Japan). QuantStudio (Thermo Fisher Scientific, Waltham, MA, USA) with TB Green^®^ Premix Ex Taq™ II (Takara, Shiga, Japan) was used for quantitative real-time PCR. The PCR reaction system was used to quantify the expression of relevant genes: pre-denaturation: 95 °C, 10 min, 1 cycle; denaturation: 95 °C, 15 s, 40 cycles; annealing extension: 60 °C, 60 s, 40 cycles. The fold changes in relative mRNA expression of each gene were calculated according to the 2^−ΔΔCT^ method and normalized by comparison with β-actin. The primer sequences are shown in Table 1.

### 2.13. Western Blotting Analysis

Colon tissue (20 mg) was lysed by adding RIPA lysis buffer containing PMSF, phosphatase inhibitor, and protease inhibitor mixture using a KZ-II high-speed tissue grinder (Servicebio, Wuhan, China). The lysed colon tissue was centrifuged at 4 °C at 1000× *g* for 10 min, and the supernatants were collected. The total protein concentrations in the supernatants were detected using the BCA protein assay kit. The denatured proteins were separated by 10% SDS-PAGE and transferred to PVDF membranes. After blocking with rapid sealing solution for 20 min at 37 °C, the membranes were incubated with β-actin (1:1000), Claudin-1 (1:2000), Occludin (1:1000), ZO-1 (1:1000), PI3K (1:1000), AKT (1:1000), NF-kappa B (1:1000), and IL-17 (1:1000) antibodies overnight at 4 °C, respectively. Then the membranes were washed with TBST and incubated with secondary antibody at 37 °C for 1 h. The chemiluminescence signals of proteins were detected with the ECL kit on the iBright™ CL1500 Imaging System. The integrated absorbance (IA) of the protein band was analyzed using ImageJ software (version No. 1.53e), and the protein relative level was normalized to β-actin (target protein IA/β-actin IA).

### 2.14. 16S rRNA Gene-Based Microbial Community Analysis

Total DNA of the fecal samples was extracted, then specific primers with Barcode were synthesized according to the full-length primer sequences, PCR amplification was performed and the products were purified, quantified and homogenized to form a sequencing library (SMRT Bell). Qualified libraries were sequenced using PacBio Sequel II. The samples were then filtered, clustered or denoised by Circular Consensus Sequencing (CCS) sequences and subjected to species annotation and abundance analysis to reveal the species composition of the samples. Alpha diversity, Beta diversity, significant species difference, correlation, and functional prediction analyses were further performed to explore the differences between samples.

### 2.15. Metabolomic Analysis

The fecal samples were ground and sonicated by adding an appropriate amount of extraction solution and magnetic beads. After centrifugation, the supernatant was collected and vacuum-dried. Then an appropriate amount of extraction solution for resolution was added for testing. An appropriate amount of extraction solution was added to the dry sample for re-dissolution and then tested on the machine. The detection platform was Waters Acquity I-Class PLUS Ultra High-Performance Liquid Chromatography (UHPLC) tandem with Waters Xevo G2-XS QTOF High Resolution Mass Spectrometer (HRMS), and the samples were detected and analyzed according to the corresponding parameters. The raw data collected using MassLynx V4.2 were subjected to data processing operations such as peak extraction and peak alignment by Progenesis QI 2.0 software, and the identification of theoretical fragments was carried out by using Progenesis QI software, the online METLIN database, public databases, and Baimaike’s self-constructed database. The Spearman correlation coefficient was used to analyze the correlation between the important metabolites and the intestinal flora.

### 2.16. Statistical Analysis

GraphPad Prism 8.0 was used for the statistical analysis. All data were expressed as mean ± SD of three independent experiments. Analysis of variance (ANOVA) was performed using IBM SPSS Statistics 22.0 to determine significant differences. The Tukey test was used when the groups had the same number of samples. Duncan’s test was used when the groups had different numbers of samples. *T*-test was used to analyze the differences between two groups. *p* < 0.05 was considered statistically significant.

## 3. Results

### 3.1. Identification of Components in TFPV

**Figure 1:**
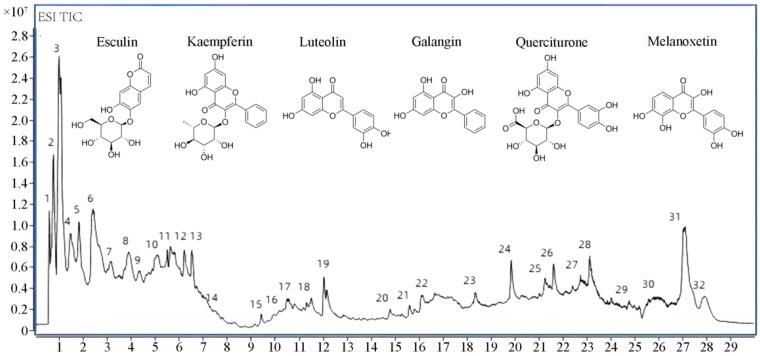
Total ion chromatography (TIC) chromatograms of TFPV.

A total of 32 components, mainly flavonoids, steroids, and terpenoids, were identified from TFPV, including kaempferin, luteolin, galangin, quercitrine, melanoxetin, etc. The total ion chromatograms (TIC) for HPLC-QTOF/MS of TFPV components are shown in Figure 1, and mass spectrometry data of each component are summarized in Table 2.

### 3.2. TFPV Can Effectively Alleviate Inflammation of LPS-Induced IPEC-J2 Cells

**Figure 2:**
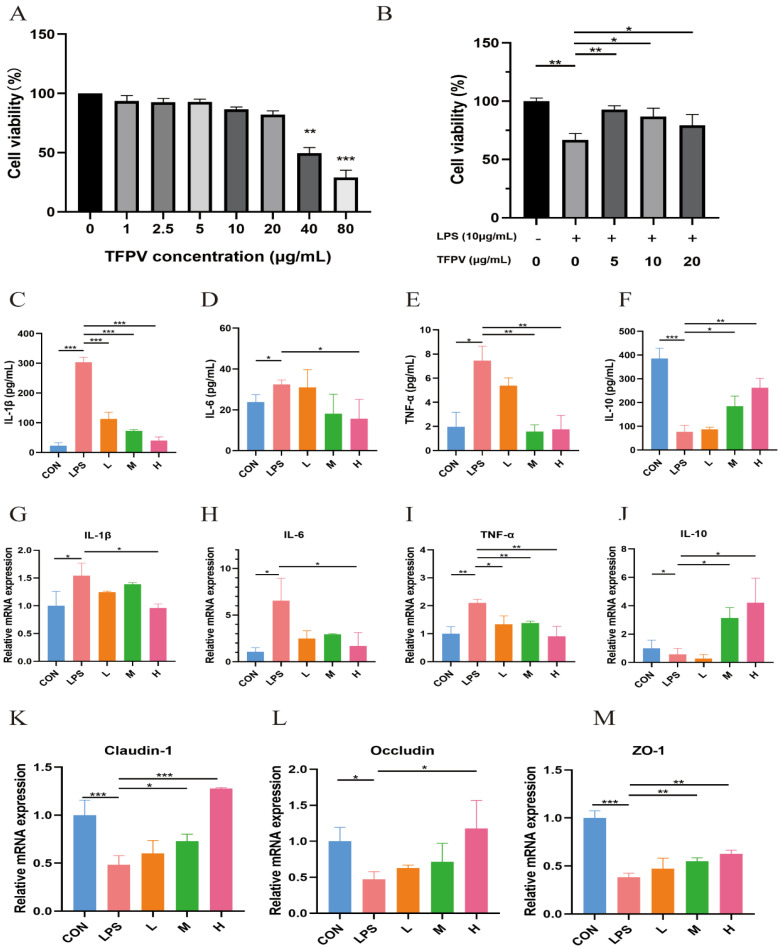
Effects of TFPV on LPS-induced IPEC-J2 cells. (**A**) Effect of TFPV on cell viability of IPEC-J2 cells. (**B**) Effect of TFPV on cell viability of LPS-induced inflammatory IPEC-J2 cells. (**C**–**F**) Effects of TFPV on the secretion levels of IL-1β, IL-6, TNF-α, and IL-10 in IPEC-J2 cells. (**G**–**J**) Effects of TFPV on mRNA expression levels of IL-1β, IL-6, TNF-α, and IL-10 in IPEC-J2 cells. (**K**–**M**) Effects of TFPV on mRNA expression levels of Claudin-1, Occludin, and ZO-1 in IPEC-J2 cells. * *p* < 0.05, ** *p* < 0.01, and *** *p* < 0.001 compared with the LPS group were considered statistically significant differences. The results are expressed as the mean ± SD. CON represents the control group; LPS represents the lipopolysaccharide group; DEX represents the positive drug group; L represents the low-dose group of TFPV; M represents the medium-dose group of TFPV; H represents the high-dose group of TFPV.

The effects of different concentrations of TFPV (1, 2.5, 5, 10, 20, 40, 80 μg/mL) on the cell viability of IPEC-J2 cells are shown in Figure 2A. When the TFPV concentration reached 40 μg/mL, the cell viability of IPEC-J2 was significantly decreased compared with the blank control group (*p* < 0.01). Therefore, three concentrations of TFPV, 5, 10, and 20 μg/mL, which had no adverse effect on the cell viability of IPEC-J2, were selected for subsequent experiments. The intracellular anti-inflammatory activity of TFPV and its effect on cellular tight junction-related genes were examined by establishing the LPS-induced inflammation model in IPEC-J2 cells. First of all, the effect of TFPV on the cell viability of LPS-induced IPEC-J2 cells is shown in Figure 2B. Compared with the blank control group, LPS significantly decreased the cell viability of IPEC-J2 cells (*p* < 0.01), while all three concentrations of TFPV significantly reversed the effect of LPS on the cell viability of IPEC-J2 cells (*p* < 0.05). Moreover, as shown in Figure 2C–F, LPS exposure significantly increased (*p* < 0.05) the concentrations of pro-inflammatory cytokines IL-1β, IL-6, and TNF-α in the supernatants of the cell culture medium, while the concentration of anti-inflammatory cytokine IL-10 was significantly decreased (*p* < 0.05). In contrast, the increase in pro-inflammatory cytokine secretion and decrease in anti-inflammatory cytokine secretion of the cells caused by LPS were significantly reversed in all dose groups of TFPV (*p* < 0.05). Detection of mRNA expression levels of IL-1β, IL-6, TNF-α, and IL-10 in cells also exhibited the same results, as shown in Figure 2G–J. It can be seen that TFPV can alleviate the inflammatory damage caused by LPS on IPEC-J2 cells by inhibiting the production of pro-inflammatory cytokines and promoting the secretion of anti-inflammatory cytokines. In addition, the detection results of tight junction-related proteins showed that LPS significantly inhibited mRNA expression levels of Claudin, Occludin, and ZO-1 in cells (*p* < 0.05), and all three doses of TFPV significantly increased their mRNA expression levels in a dose-dependent trend (Figure 2K–M). Therefore, TFPV has a certain protective effect on cellular tight junctions.

### 3.3. TFPV Improves LPS-Induced Intestinal Inflammation in Mice by Enhancing the Intestinal Barrier Function

**Figure 3:**
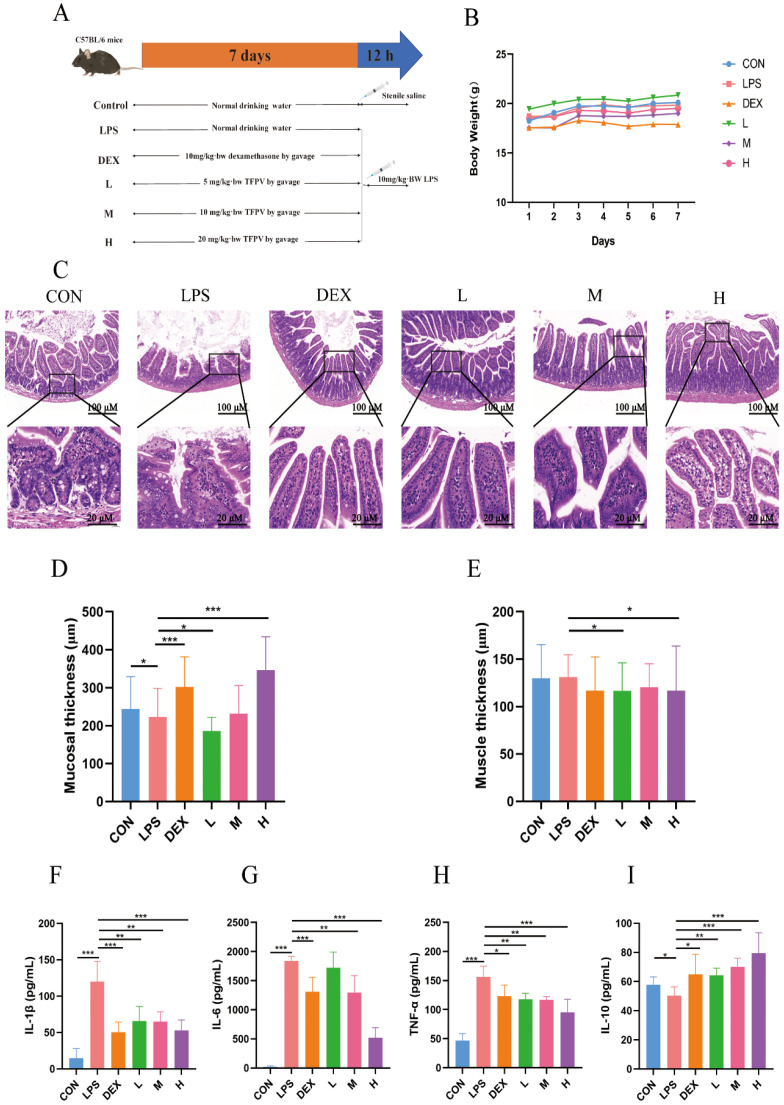
Effects of TFPV on LPS-induced body weight, intestinal morphology, muscle layer, mucosal layer, and inflammatory factors. (**A**) experimental design. (**B**) Weight changes. (**C**) Pathological damage. (**D**) Mucosal thickness. (**E**) Muscle thickness. (**F**–**I**) Effects of TFPV on the levels of IL-1β, IL-6, TNF-α, and IL-10 in the serum of mice of each group. * *p* < 0.05, ** *p* < 0.01, and *** *p* < 0.001 compared with the LPS group were considered statistically significant differences. The results are expressed as mean ± SD (*n* = 8). CON represents the control group; LPS represents the lipopolysaccharide group; DEX represents the positive drug group; L represents the low-dose group of TFPV; M represents the medium-dose group of TFPV; H represents the high-dose group of TFPV.

The experiment design is shown in Figure 3A, and the body weight of mice in each treatment group was counted (Figure 3B). Previous studies have reported that intestinal immune cells, such as macrophages and T cells, secrete large amounts of inflammatory factors such as TNF-α, IL-1β, and IL-6 under the stimulation of LPS, leading to increased permeability of intestinal epithelial cells and disruption of the intestinal mucosal barrier, thus making it easier for bacteria and toxins to enter the intestinal wall and trigger intestinal inflammation [29]. In this study, the effects of TFPV on intestinal inflammation in mice were investigated by establishing the LPS-induced intestinal inflammation model in mice. The pathological sections of the jejunum of mice in each treatment group are shown in Figure 3C. A small number of mucosal epithelial cells with degeneration and necrosis, nucleolysis, and inflammatory cell infiltration were observed in the jejunum tissues of mice in the LPS group. The jejunal tissues of mice in the blank control group and the TFPV group at each dose, on the other hand, showed no obvious pathological changes.

By measuring the thickness of intestinal mucosa and the muscle layer of mice in each group, it was found that compared with the control group, LPS treatment significantly reduced the thickness of intestinal mucosa (Figure 3D, *p* < 0.05), while the thickness of the muscle layer showed no significant change compared with the control group (Figure 3E, *p* > 0.05). The intestinal mucosal layer thickness of mice treated with DEX and TFPV at high doses was significantly higher compared with that of the LPS group (Figure 3D, *p* < 0.001), suggesting that the high dose of TFPV could protect the intestinal mucosa by restoring the reduced intestinal mucosal layer thickness caused by LPS in mice. It can be concluded that TFPV can alleviate intestinal tissue and intestinal mucosal damage caused by LPS in mice. In addition, the detection results of pro- and anti-inflammatory cytokines in serum of mice in each treatment group were consistent with the results of the cellular assay (Figure 3F–I), that is, LPS significantly increased the serum concentrations of pro-inflammatory cytokines IL-1β, IL-6, and TNF-α (*p* < 0.001), and significantly decreased the serum concentration of anti-inflammatory cytokine IL-10 (*p* < 0.05). Three doses of TFPV, on the other hand, significantly reversed the increase in pro-inflammatory cytokines and decrease in anti-inflammatory cytokines in the serum of mice caused by LPS (*p* < 0.05).

**Figure 4:**
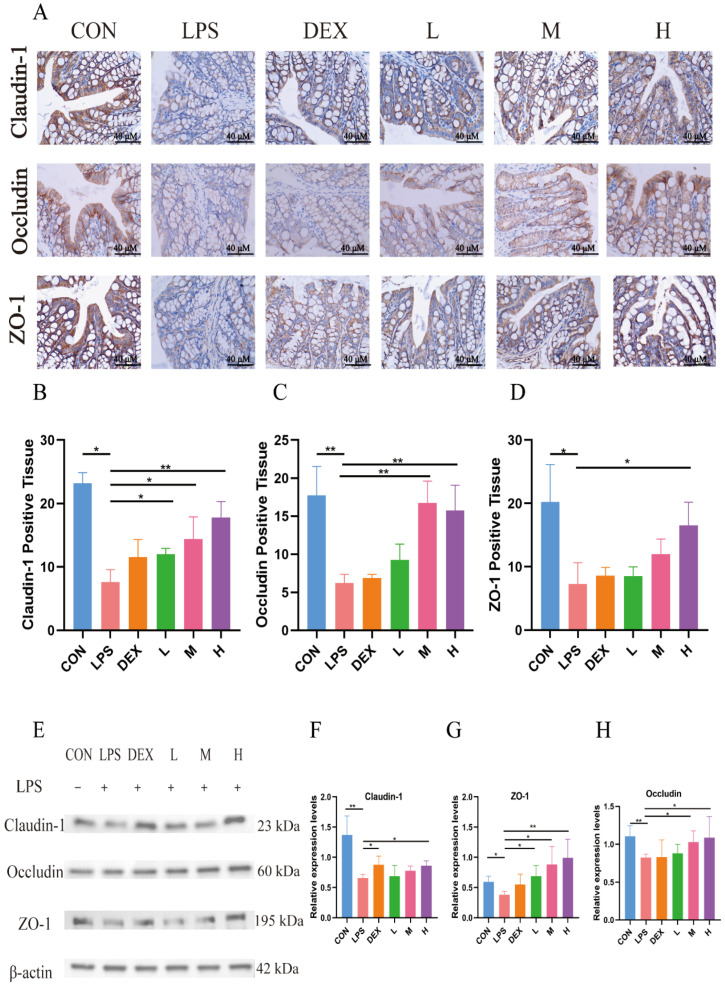
TFPV improved the expression levels of tight junction proteins induced by LPS in mice. (**A**) The positive expression of Claudin-1, Occludin, and ZO-1 in colonic tissues. The scale bar was 40×. (**B**–**D**) Statistical results of the proportion of positive areas. (**E**) Western blot protein bands of Claudin-1, Occludin, and ZO-1. (**F**–**H**) The histograms of band intensity analysis of Claudin-1, Occludin, and ZO-1. * *p* < 0.05, ** *p* < 0.01 ompared with the LPS group were considered statistically significant differences. The results are expressed as mean ± SD (*n* = 3). CON represents the control group; LPS represents the lipopolysaccharide group; DEX represents the positive drug group; L represents the low-dose group of TFPV; M represents the medium-dose group of TFPV; H represents the high-dose group of TFPV.

The expressions of tight junction proteins Claudin-1, Occludin, and ZO-1 in mouse colon tissues were detected by IHC and WB, respectively, and the results are shown in Figure 4. Compared with the blank control group, LPS significantly down-regulated the positive expression of Claudin-1, Occludin, and ZO-1 and their protein expression levels in colonic tissues of mice (*p* < 0.05). Whereas all three doses of TFPV could reverse the effects of LPS on the expression of Claudin-1, Occludin, and ZO-1 to varying degrees, among which, the effect of the high dose of TFPV was the most significant, which significantly up-regulated the positive expression and protein expression levels of the three tight junction proteins (*p* < 0.05). The above results indicated that TFPV can play a role in protecting the intestines of LPS-induced enteritis mice by regulating the levels of inflammatory cytokines, restoring the reduced thickness of the intestinal mucosal layer caused by LPS, and enhancing the intestinal barrier function.

After comprehensive analysis of the above data, it was found that low, medium, and high concentrations of TFPV had therapeutic effects on LPS-induced intestinal inflammation in mice, but the effect of the high-dose group was significantly better than that of the low- and medium-dose groups. Therefore, the high-dose group was selected for subsequent experiments to further investigate the potential effects of TFPV.

### 3.4. Targets Prediction of TFPV for UC by Network Pharmacology

**Figure 5:**
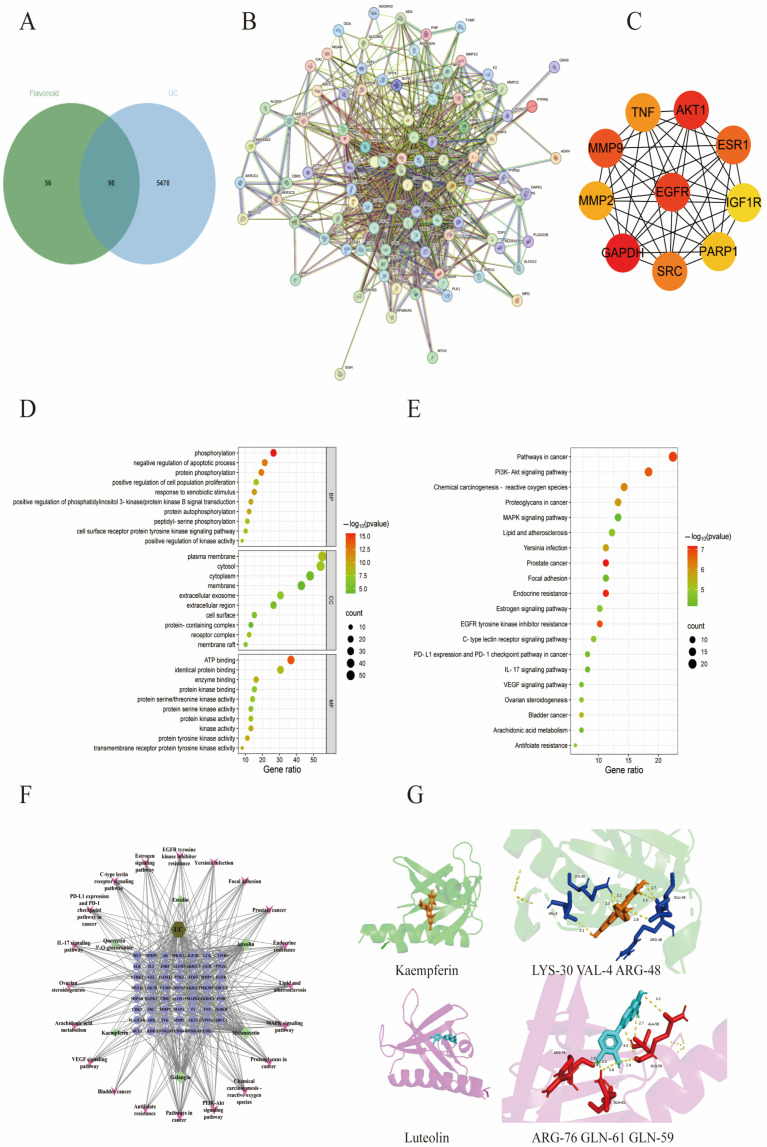
Network pharmacological analysis of the effect of TFPV on UC. (**A**) Venn diagram of the interactions between candidate targets of TFPV and UC-related targets. (**B**) The protein–protein interaction (PPI) network of TFPV’s action on UC. (**C**) The screened core targets. (**D**) Bubble chart of GO enrichment analysis. (**E**) Bubble chart of KEGG enrichment analysis. (**F**) The integrated visualization network diagram of component–disease–pathway. (**G**) Molecular docking results of AKT1 with kaempferol and luteolin.

Detailed chemical information on the six flavonoid components of TFPV, kaempferin, luteolin, galangin, quercitrine, melanoxetin, and esculin (precursors of flavonoids), was obtained from the PubChem database (https://pubchem.ncbi.nlm.nih.gov/), and the action targets (probability > 0.1) of each component were retrieved from the Swiss target prediction database (http://www.swisstargetprediction.ch/). A total of 154 targets was obtained for subsequent analysis. The intersection of 5571 UC-related targets queried in the Genecards database (https://www.genecards.org) using “ulcerative colitis” as the keyword with 154 targets of TFPV was obtained through a Venn diagram. As shown in Figure 5A, a total of 98 intersecting targets associated with TFPV action on UC were eventually obtained.

The results of protein interactions were visualized using the Cytoscape application (Cytoscape 3.7.2). The specific information of interactions between targets was obtained after calculation by the MCC algorithm of the plug-in CytoHubba. As shown in Figure 2B, nodes represent target proteins, and edges represent interactions between proteins. The higher the network connectivity, the tighter the relationship between proteins. The larger the protein knot, the higher the binding degree. Based on the specific information of protein interactions, the top five core targets were screened as GAPDH, AKT1, EGFR, MMP9, and ESR1 (Figure 5C). The results of GO enrichment analysis and KEGG enrichment analysis were visualized using bubble plots. Figure 5D shows the top ten enriched biological processes (BP), cellular components (CC), and molecular functions (MF) associated with TFPV action on UC, respectively. The bubble map of KEGG enrichment analysis selected the pathways with statistical significance and the number of enriched genes greater than six according to the *p*-value for drawing, as shown in Figure 5E. The KEGG enrichment results indicated that the signaling pathways associated with TFPV action on UC were mostly inflammation-related pathways, such as the PI3K-AKT signaling pathway, IL-17 signaling pathway, etc. The component–disease–pathway network diagram constructed based on the results of network pharmacology analysis is shown in Figure 5F.

In addition, the literature reports have indicated that some of the components in TFPV, including kaempferol and luteolin, have an alleviating effect on UC. Therefore, kaempferol and luteolin were selected in this study for molecular docking with one of the core targets screened by network pharmacology, which was AKT (aliases: AKT, PKB, etc.). The binding energy in molecular docking is usually within a specific range that can help assess the strength of the interaction between the small molecule ligand and the target protein. A lower value of binding energy usually indicates a stronger interaction between the ligand and the target protein [30]. As shown in Figure 5G, kaempferin had a binding energy of −9.33 kcal/mol to protein AKT and could form hydrogen bonds with residues LYS-30, VAL-4, ARG-48, and GLU-49. Luteolin had a binding energy of −6.85 kcal/mol to protein AKT1 and could form hydrogen bonds with residues ARG-76, GLN-61, GLN-59, and ALA-58. The above results indicated that kaempferin and luteolin could match well with the active pockets of AKT.

### 3.5. TFPV Alleviates DSS-Induced UC in Mice by Regulating Intestinal Inflammatory Levels and Enhancing Intestinal Barrier Function

**Figure 6:**
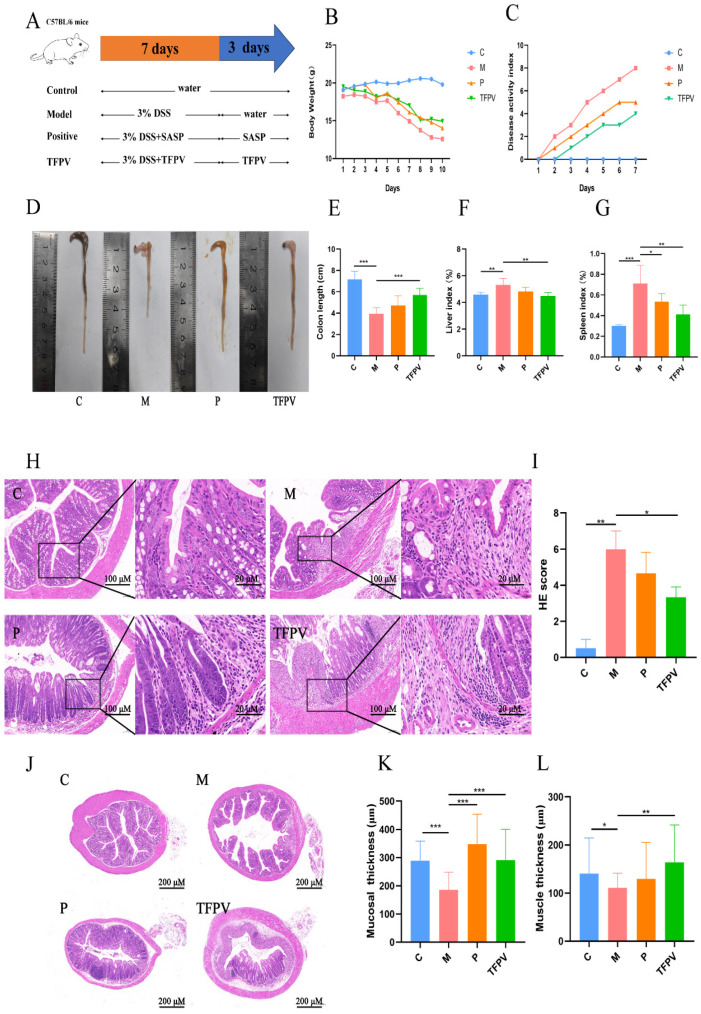
Effect of TFPV on DSS-induced UC in mice. (**A**) Experimental design. (**B**) Body weight changes. (**C**) Disease activity index (DAI) score. (**D**,**E**) The colon length. (**F**) Liver index. (**G**) Spleen index. (**H**) Pathological observation of colon tissues (HE, Scale bars,100 µm). (**I**) Pathological scores of colon tissues. (**J**–**L**) Thickness of the colon mucosal layer and the muscle layer. * *p* < 0.05, ** *p* < 0.01, and *** *p* < 0.001 compared with the M group were considered statistically significant differences. The results are expressed as mean ± SD (*n* = 10). C represents the control group; M represents the DSS group; P represents the positive control group; TFPV represents the TFPV treatment group.

In order to further investigate the potential therapeutic effect of TFPV on UC, the DSS-induced UC model in mice was established in this study, and the experimental protocol is shown in Figure 6A. Changes in body weight and disease activity index (DAI) can reflect the health status of mice. As can be seen in Figure 6B,C, TFPV treatment alleviated the weight loss and elevated DAI index caused by DSS in mice. Measurements of the colon length of mice in each treatment group showed that the colon length of mice in the model group was significantly lower than that of the blank control group (Figure 6D,E, *p* < 0.001), whereas the colon lengths of mice in the positive group and the TFPV group were significantly higher than that of the model group (Figure 6D,E, *p* < 0.001). Meanwhile, measurement of organ indices showed that DSS induction had significant effects on liver and spleen indices in mice. That is, DSS significantly increased the liver and spleen indices of mice compared to the blank control group, while TFPV significantly reversed the effects of DSS on the liver and spleen indices of mice (Figure 6F,G, *p* < 0.01).

In addition, the pathological sections and pathological scores of the colon tissues of mice also reflected the therapeutic effect of TFPV on DSS-induced UC in mice; that is, the mucosal layer of the colonic tissues of mice in the model group was significantly degenerated and necrotic, with mucosal epithelial exfoliation, blurred structure, and inflammatory cell infiltration (Figure 6H,I, *p* < 0.01), whereas the treatment of TFPV significantly ameliorated the above pathologic changes (Figure 6H,I, *p* < 0.05). It has been reported that DSS-induced triggered inflammatory response damages intestinal epithelial cells, induces apoptosis, and leads to tissue loss, as well as contributes to reduced intestinal mucosal thickness, intestinal mucosal damage, and intestinal inflammation. The effect of DSS on the intestinal muscle layer is even more complex, as the inflammation caused by DSS may result in adhesion and hypertrophy of the intestinal muscle layer [31]. Measurements of the thickness of the colonic mucosal layer and muscle layer of mice in each treatment group were shown in Figure 6J,K, which showed that TFPV significantly ameliorated the DSS-induced reduction in the thickness of the colonic mucosal and muscular layers of mice (*p* < 0.01).

**Figure 7:**
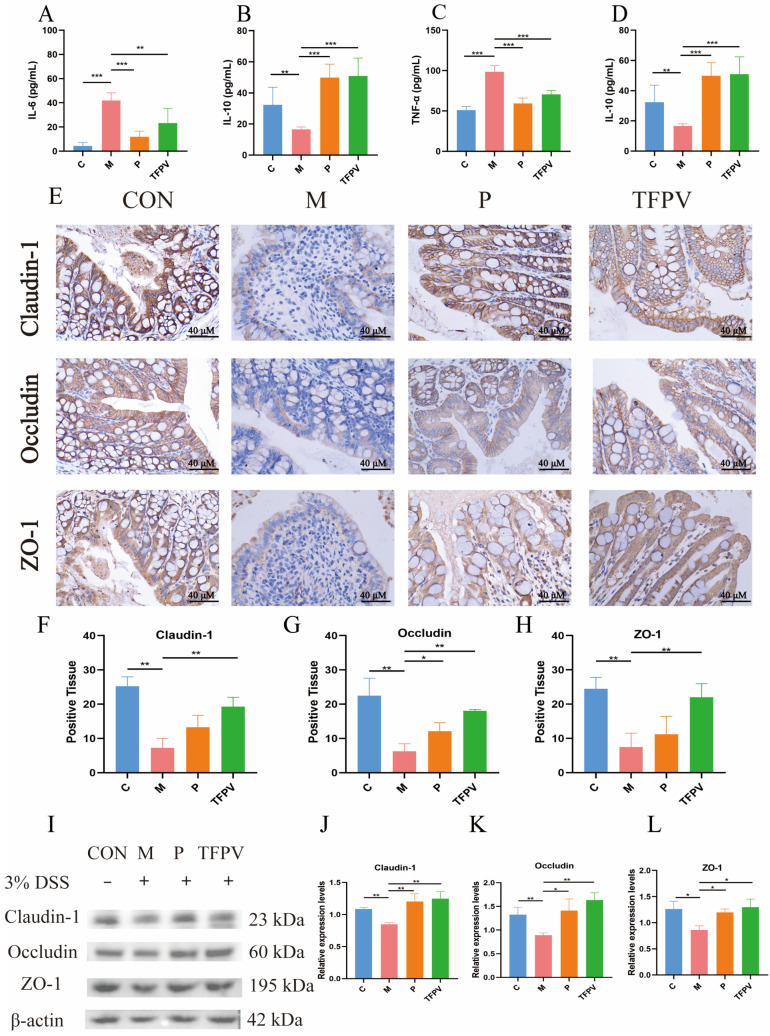
TFPV ameliorated DSS-induced UC in mice. (**A**–**D**) Effects of TFPV on the levels of TNF-α, IL-1β, IL-6, and IL-10 in the serum of mice in each treatment group (*n* = 8). (**E**) The positive expression of Claudin-1, Occludin, and ZO-1 in colonic tissues (*n* = 3). Scale bar was 40×. (**F**–**H**) Statistical results of the proportion of positive areas. (**I**) Representative protein bands of Claudin-1, Occludin, and ZO-1, (*n* = 3). (**J**–**L**) The histograms of band intensity analysis of Claudin-1, Occludin, and ZO-1. * *p* < 0.05, ** *p* < 0.01 and *** *p* < 0.001 compared with the M group were considered statistically significant differences. The results are expressed as mean ± SD. C represents the control group; M represents the DSS group; P represents the positive control group, TFPV represents the TFPV treatment group.

Subsequently, the detection of cytokine levels demonstrated that TFPV significantly reversed the increase in the levels of pro-inflammatory cytokines TNF-α, IL-1β, and IL-6 and the decrease in the levels of anti-inflammatory cytokine IL-10 in the serum of mice caused by DSS induction (Figure 7A–D, *p* < 0.05). In addition, the expression of tight junction proteins was examined by IHC. As can be seen in Figure 7E–H, the induction of DSS significantly down-regulated the positive expression of Claudin-1, Occludin, and ZO-1 proteins in colon tissues of mice compared to the blank control group (*p* < 0.01), while TFPV significantly ameliorated the inhibition of the expression of these three types of tight junction proteins in mouse colon tissues by DSS (Figure 7E–H, *p* < 0.01). The expression levels of Claudin-1, Occludin, and ZO-1 proteins continued to be detected by WB assay, and the results were consistent with those of IHC detection (Figure 7I–L). These data suggest that TFPV can alleviate DSS-induced UC in mice by regulating the levels of inflammatory cytokines in serum, inhibiting the reduction in the thickness of the intestinal mucosal and muscular layers caused by DSS, and enhancing intestinal barrier function. Furthermore, to further explore the mechanism of action of TFPV in alleviating DSS-induced UC in mice, the results of network pharmacological analysis were validated.

**Figure 8:**
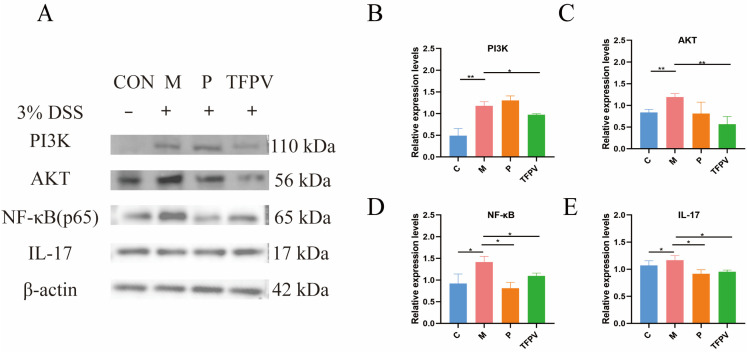
TFPV ameliorated DSS-induced UC in mice. (**A**) Representative protein bands of PI3K, AKT, NF-κB, and IL-17 (*n* = 3). (**B**–**E**) The histograms of bands intensity analysis of PI3K, AKT, NF-κB, and IL-17. * *p* < 0.05, ** *p* < 0.01 and *** *p* < 0.001 compared with the M group were considered statistically significant differences. The results are expressed as mean ± SD. C represents the control group; M represents the DSS group; P represents the positive control group; TFPV represents the TFPV treatment group.

It has been reported that the PI3K/AKT/NF-κB signaling pathway plays a key role in the inflammatory response [32]. As illustrated in Figure 8A–D, TFPV significantly reduced the protein expression levels of PI3K, AKT, and NF-κB (*p* < 0.05), indicating that TFPV effectively alleviates inflammation through the inhibition of the PI3K/AKT/NF-κB signaling pathway. Furthermore, TFPV demonstrated a significant inhibitory effect on the expression of IL-17 protein and its associated downstream inflammatory mediators (Figure 8A,E, *p* < 0.05), which contributed to the mitigation of colonic inflammation and tissue damage. This finding indicates that TFPV possesses anti-inflammatory properties through the suppression of the IL-17 signaling pathway. To sum up, TFPV may confer its protective effects through the synergistic interaction among multiple targets and pathways. Specifically, TFPV effectively regulates the PI3K/AKT/NF-κB signaling pathway, preventing its aberrant activation, which in turn diminishes the nuclear translocation and activation of NF-κB, leading to a reduction in the expression of downstream pro-inflammatory factors. Meanwhile, TFPV inhibited the expression and signaling of the pivotal inflammatory mediator IL-17, thereby reducing the excessive inflammatory response of immune cells. Collectively, these regulatory actions facilitated the recovery and enhancement of intestinal barrier function, while alleviating the inflammatory damage and structural disarray associated with DSS-induced UC in mice. Consequently, TFPV exhibits pronounced anti-inflammatory and intestinal protective effects through the modulation of the PI3K/AKT/NF-κB and IL-17 signaling pathways.

### 3.6. TFPV Enhances the Diversity of the Intestinal Microbiota and Reverses the DSS-Induced Dysbiosis in Mice

**Figure 9:**
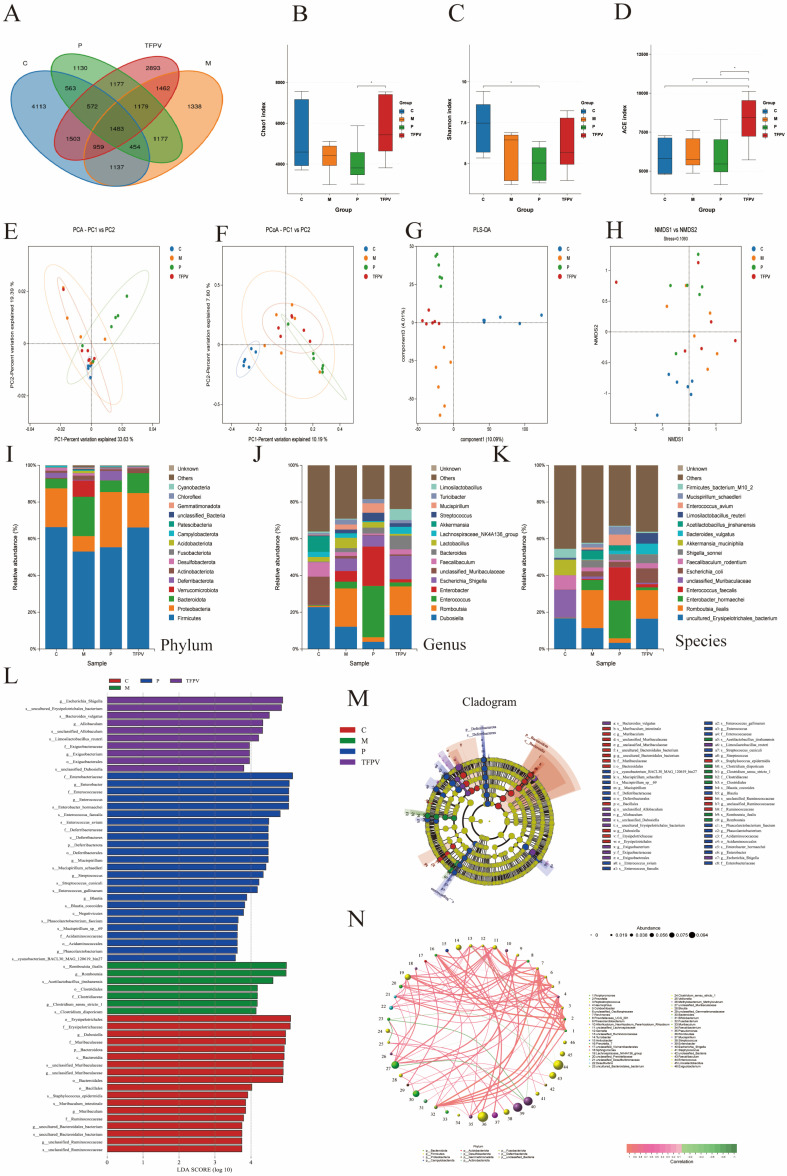
The diversity of microbiota content in the cecum was analyzed by third-generation microbial 16S rDNA analysis. TFPV was found to regulate DSS-induced dysbiosis in the gut microbiota of mice. (**A**) The Venn diagram shows a comparison of common species among four groups. (**B**) Chao 1 index. (**C**) Shannon index. (**D**) ACE index. (**E**) PCA analysis. (**F**) PCoA analysis. (**G**) OPLS-DA analysis. (**H**) NMDS analysis. (**I**–**K**) Microbial community structure at the phylum, genus, and species levels of each treatment group. (**L**) Histogram of differential microbial distribution based on LDA score. (**M**) The enriched microbial community evolution map generated by LEfSe analysis. (**N**) Correlation network analysis. The results are expressed as mean ± SD (*n* = 6). C represents the control group; M represents the DSS group; P represents the positive control group; TFPV represents the TFPV treatment group.

In order to investigate the effect of TFPV on the intestinal flora of mice with DSS-induced UC, the cecal contents of mice from each treatment group were collected for 16S rRNA gene sequencing analysis. Firstly, Venn diagrams can be used to display the number of common and unique features among samples, from which the overlap of features between samples can be visualized. As shown in Figure 9A, a total of 21,140 OTUs were detected in the samples of the four treatment groups, among which 1483 OTUs were shared. Secondly, the Alpha diversity of the samples in each group was assessed using the Chao 1 index, the Shannon index, and the ACE index, and all of the indices showed a similar trend, that is, the DSS treatment reduced the number and species diversity of microbial species in the cecal contents of the mice to a certain extent, while TFPV treatment reversed the effect of DSS on microbial community abundance, but the difference was not significant (*p* > 0.05). Subsequently, differences in the microbiota structure of the samples from each group were assessed using Beta diversity analysis. Among them, Principal Component Analysis (PCA) and Principal Coordinate Analysis (PCoA) were used to determine the overall differences in microbial communities between groups [33], while Non-Metric Multi-Dimensional Scaling (NMDS) and orthogonal projections to latent structures-discriminant analysis (OPLSA-DA) score charts directly reflected inter-group and intra-group differences. As shown in Figure 9E–H, there were significant differences in the microbial communities of cecal contents samples of mice in the four treatment groups, indicating that different treatments had significant effects on the structure of intestinal microbial communities of mice.

The results of the analysis of microbial community structure at the phylum, family, and genus levels were visualized using histograms (Figure 9I–K), where each histogram represents a sample, different colors are used to distinguish between taxonomic units, and the vertical coordinates indicate the relative abundance of each taxonomic unit. As shown in Figure 9I–K, the relative abundance of Firmicutes decreased in the samples of the DSS group, while the relative abundance of Bacteroidetes increased. However, after treatment with TFPV, these two phyla showed opposite trends. Line Discriminant Analysis Effect Size (LEfSe) analysis was performed to identify species that differed significantly between groups (LDA score > 3.5), and the results are shown in Figure 9L,M. A total of 59 taxonomic groups were obtained at the phylum and genus levels from the four groups, including 18 taxonomic units in Group C, 7 in Group M, 24 in Group P, and 10 in group TFPV. The most diverse taxonomic units in abundance in group C were *Erysipelotrichales, Erysipelotrichaceae*, and *Dubosiella*. The taxonomic units with the largest differences in abundance in group M were *Romboutsia ilealis*, *Romboutsia*, and *Acctilactobacillus jinshanensis*. The taxonomic units with the largest abundance differences in group P were *Enterobacteriaceae*, *Enterobacter*, and *Enterococcus*. The taxonomic units with the largest abundance differences in group TFPV were *Escherichia Shigella*, *Erysipelotricnales bacterium*, and *Bacteroides vulgatus*. It has been reported that, based on correlation network analysis, the co-existence relationship and important pattern information of species in the same environment can be obtained, and the mechanism of phenotypic differences between samples can be further explained. Therefore, Spearman rank correlation analysis based on the abundance and variation of each species in the four groups of samples was conducted in this study, and correlation networks were constructed by selecting data with correlations greater than 0.1 and *p*-values less than 0.05. As can be seen from Figure 9N, bacterioidetes and firmicutes had the highest correlation among all samples.

### 3.7. TFPV Regulates Purine Metabolism and Reduces Uric Acid Levels in Mice with DSS-Induced UC

**Figure 10:**
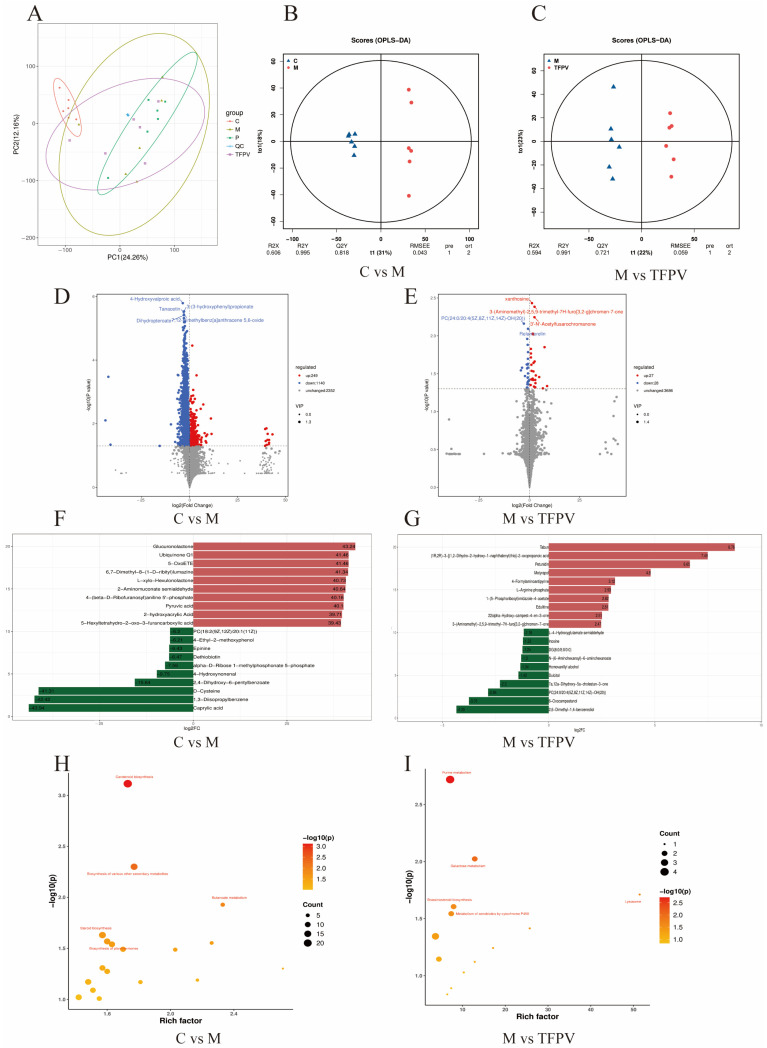
Effect of TFPV on metabolomics in fecal contents in mice with DSS-induced UC. (**A**) PCA diagram of metabolites. (**B**,**C**) Plots of OPLS-DA scores between group C and group M and between group M and group TFPV. (**D**,**E**) Volcanic diagrams of differential metabolites. (**F**,**G**) Histogram of the difference multiples of the top 10 up-regulated and down-regulated differential metabolites between group C and group M, and between group M and group TFPV. (**H**,**I**) Bubble plots of metabolic pathways enriched in differential metabolites between group C and group M, and between groups M and group TFPV. C represents the control group; M represents the DSS group; P represents the positive control group; TFPV represents the TFPV treatment group.

To explore the effects of TFPV on metabolites and metabolic pathways in DSS-induced UC mice, the present study conducted non-targeted metabolomics analysis on fecal content samples (colon) of mice in the four experimental groups based on the LC-QTOF platform, and a total of 3741 metabolites were identified. Statistical analysis of the test samples by PCA showed that the metabolic profiles of the four groups were highly differentiated, with good reproducibility of the samples within each group (Figure 10A). It has been reported that PCA, although effective in extracting key information, is insensitive to variables with low correlation, and orthogonal projections to latent structures-discriminant analysis (OPLS-DA) can solve this problem. The OPLS-DA model scores between group C and group M, as well as between group M and group TFPV are shown in Figure 10B,C. The R^2^Y values of the two models were greater than 0.9, and the Q^2^Y values were greater than 0.5, indicating that the two models established were stable and reliable and could be used for subsequent screening of differential metabolites.

**Figure 11:**
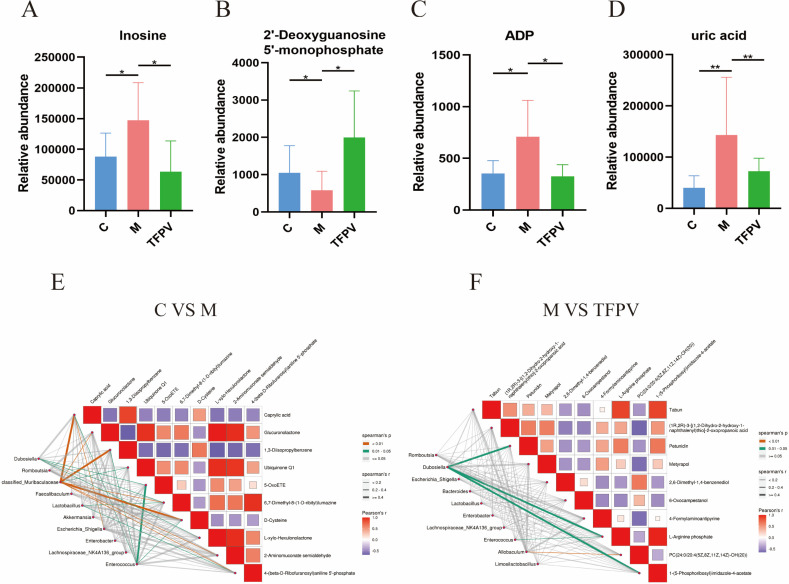
Correlation analysis of metabolites and intestinal microbiota in mice of different experimental groups. (**A**–**D**) Relative abundance of inosine, dGMP, ADP, and uric acid in fecal contents of mice from different treatment groups. (**E**,**F**) Heat map of the correlation between metabolites and gut microbiota composition of mice between different treatment groups. Different colors represent the magnitude of the Pearson correlation coefficient, and the closer the absolute value is to 1, the higher the correlation is. Red represents positive correlation, while blue represents negative correlation, with darker colors indicating stronger correlation. * *p* < 0.05, ** *p* < 0.01 compared with the M group were considered statistically significant differences. The results are expressed as mean ± SD (*n* = 6). C represents the control group; M represents the DSS group; P represents the positive control group; TFPV represents the TFPV treatment group.

Further, Variable Importance in Projection (VIP) ≥ 1 and fold change (FC) ≥ 1 were used as screening criteria for metabolite screening. The volcano plots of differential metabolites are shown in Figure 10D,E. A total of 1389 differential metabolites were screened between group C and group M, of which 249 were up-regulated and 1140 were down-regulated, and a total of 55 differential metabolites were screened between group M and group TFPV, of which 27 were up-regulated and 28 were down-regulated. After qualitative and quantitative analysis of the detected metabolites, the difference multiples of the differential metabolites were treated by log conversion. The top 10 differential metabolites that were up-regulated and down-regulated in group C compared to group M and group M compared to group TFPV were shown in Figure 10F,G, respectively. Complex metabolic reactions and their regulation in organisms do not occur in isolation but are often formed by different genes and proteins into complex pathways and networks. To more systematically understand the effects of TFPV on metabolic pathways in DSS-induced UC mice, the KEGG database was used to annotate screened differential metabolites. The bubble diagrams of the KEGG enrichment factor of differential metabolites between group C and group M and between group M and TFPV are shown in Figure 10H,I. It can be seen that the differential metabolites in the purine metabolism pathway were enriched to the highest extent between groups M and TFPV. Analysis of the important intermediates and products of purine metabolism, inosine, 2′-Deoxyguanosine 5′-monophosphate (dGMP), adenosine diphosphate (ADP), and uric acid, revealed that the relative abundance of inosine, ADP, and uric acid was significantly increased in the model group as compared to that of the blank control group (Figure 11A,B, *p* < 0.05) and a significant decrease in the relative abundance of dGMP (Figure 11C, *p* < 0.05). These results indicated that TFPV alleviates inflammation in DSS-induced UC mice by regulating purine metabolism to restore normal uric acid levels.

In addition, according to the Spearman correlation coefficient between differential metabolites and gut microbiota composition, significant correlations were found between group C and group M in caproic acid, 1,3-diisopropylbenzene, D-cysteine, and *Muribaculaceae* (Figure 11E). While (1R, 2R)-3-[(1,2-dihydro-2-hydroxy-1-naphthalenyl)thio]-2-oxopropanoic acid, 4-formylaminoantipyrine, 1-(5-phosphoribosyl)imidazole-4-acetate, and *Dubosiella* between group M and group TFPV had significant correlations (Figure 11F).

## 4. Discussion

Plant extracts containing bioactive compounds like polysaccharides, flavonoids, and alkaloids are being increasingly recognized as promising therapeutic candidates for UC. For instance, studies have demonstrated the efficacy of *Aqueous Moringa oleifera* leaf extract and baicalin in ameliorating UC symptoms [34]. Among the numerous active components of plant extracts, flavonoids have been extensively studied due to their multifunctional biological activities.

PV, a traditional Chinese herb utilized for treating dysentery and gastrointestinal disorders [35], contains flavonoids, volatile oils, and saponins known for their anti-inflammatory, antibacterial, and antioxidant properties [36]. Despite this, the therapeutic effects and mechanisms of TFPV in UC have not been thoroughly investigated. In this study, TFPV was extracted and purified, with LC-MS analysis identifying key components such as esculin, luteolin, melanoxetin, galangin, and kaempferin. Esculin exhibited in vivo anti-inflammatory activity by regulating TNF-α and IL-6 production in LPS-induced murine macrophages [37]. In addition, galangin, kaempferin [38], luteolin [39], and quercetin [40] were found to protect against DSS-induced UC in mice by reducing intestinal inflammation and modulating gut microbiota [41].

Collectively, prior research on the primary components of TFPV and their anti-UC effects [42] suggests that TFPV exerts robust in vitro and in vivo anti-inflammatory activity, positioning it as a promising plant extract for the treatment of UC.

Research has shown that LPS can disrupt intestinal structure, damage intestinal villi, and cause intestinal barrier dysfunction [43]. PV has been shown to inhibit LPS-induced inflammation of macrophages by inducing heme oxygenase-1 and activating the Nrf2 signaling pathway [44]. Therefore, in the present study, the model of LPS-induced inflammation in IPEC-J2 cells as well as the model of LPS-induced intestinal inflammation in C57BL/6 mice were established in order to investigate the effects of TFPV on LPS-induced intestinal inflammatory responses and intestinal barrier dysfunction in vivo and in vitro. The experiment results of cell and mouse models showed that the expression of pro-inflammatory cytokines TNF-α, IL-1β, and IL-6, and anti-inflammatory cytokine IL-10 were significantly increased and decreased in the LPS group compared with the blank control group, respectively, which was consistent with previous studies [45]. TFPV treatment, on the other hand, significantly reversed the elevation of pro-inflammatory cytokine expression and the decrease in anti-inflammatory cytokine expression caused by LPS. Overexpression of inflammatory factors such as TNF-α and IL-1β has been reported to cause epithelial barrier damage, epithelial cell apoptosis, decreased epithelial cell barrier function, and exacerbated intestinal inflammation. HE pathological analysis of the mouse model showed that the intestinal villi of mice in the LPS group were fragmented and atrophied, and the intestinal epithelial cells were misaligned. In contrast, after treatment with TFPV, the intestinal villi of mice were tightly arranged, and the intestinal structure was returned to normal. The thickness of the intestinal mucosa and muscle layers has been shown to correlate with the health of the intestinal barrier [46]. The experimental results of this study showed that TFPV restored the reduction in the thickness of the intestinal mucosal layer in mice caused by LPS. In addition, numerous studies have demonstrated that the intestinal epithelial barrier is maintained by a series of tight junctions (TJs) composed of transmembrane proteins (Claudin, Occludin, ZO-1, etc.), which play a crucial role in maintaining the integrity and function of the intestinal tract. These transmembrane proteins form a closed barrier between epithelial cells, preventing the free passage of intercellular substances [47]. Disruption or loss of tight connections can lead to the phenomenon of “leaky gut” and increase intestinal permeability, which is closely related to various intestinal diseases such as inflammatory bowel disease and irritable bowel syndrome [48]. Therefore, healthy tight junctions are essential for the integrity of the intestinal barrier and the maintenance of normal intestinal function. LPS is one of the inducements that cause intestinal tight junction injury and increase the permeability of intestinal epithelial cells. The results of IHC and WB in this study showed that LPS significantly reduced the positive expression and protein expression levels of TJ proteins, Claudin, Occludin, and ZO-1 in colonic tissues, while TFPV alleviated this trend. According to the above results, it can be seen that TFPV can inhibit the influence of LPS on the expression of pro-inflammatory and anti-inflammatory cytokines, restore the reduction of intestinal mucosal thickness caused by LPS, alleviate the intestinal tight junction damage caused by LPS, and enhance the intestinal barrier function, thus having a good effect on the resistance of LPS-induced intestinal inflammation in vivo and in vitro.

Network pharmacology is an emerging method for exploring therapeutic targets of drugs [49,50], which was used in this study to investigate the potential targets of TFPV in the treatment of UC. Six components of TFPV with high GI absorption scores were selected for network pharmacology analysis, and 98 targets intersecting with action targets of UC were screened. Among them, the top five core targets with the highest protein binding degree were GAPDH, AKT1, EGFR, MMP9, and ESR1. Further enrichment analysis of the 98 cross-targets showed that the biological process (BP) associated with TFPV treatment of UC mainly involved phosphorylation and negative regulation of the apoptotic process, and the cellular component (CC) mainly involved the plasma membrane and cytosol. Molecular function (MF) enrichment results indicated that most of these targets were related to ATP binding and identical protein binding. Moreover, molecular docking simulations verified the binding ability of the main components of TFPV, kaempferin and luteolin, to the core gene AKT1, and the results showed that these two components can be stabilized as ligands into the protein pockets of AKT1. The results of KEGG enrichment analysis indicated that the pathway in cancer and the PI3K/AKT signaling pathway were the two signaling pathways with the highest enrichment of TFPV targets acting on UC. Numerous studies have shown that activation of the PI3K/AKT signaling pathway can participate in inflammatory responses and regulate immune responses [51]. Therefore, this pathway may be one of the key pathways of TFPV for the treatment of UC. Furthermore, these two pathways are associated with tumorigenesis and cellular signal transduction, respectively, so TFPV may simultaneously affect tumor development and cellular signal transduction processes, which suggests that TFPV may have a multi-target and multi-pathway pharmacological mechanism of action.

UC is a lifelong autoimmune disease that can lead to a number of dangerous complications if left untreated. An increasing number of people worldwide are affected by this disease, and patients typically need lifelong medication [7]. However, long-term use of drugs can lead to reduced clinical efficacy as well as serious side effects. In recent years, natural products have shown great potential in the treatment of UC, especially in terms of anti-inflammatory, antioxidant, and intestinal barrier protection [52]. With the deepening of research on these natural products, more effective natural therapies may be available for the prevention and treatment of UC in the future [53]. As a traditional Chinese herbal medicine, PV has been used for centuries in China for the treatment of enteritis and dysentery. However, there is no clear information about its efficacy and mechanism of action on UC. Therefore, the present study further established a DSS-induced UC mouse model to investigate the therapeutic effect and the related mechanisms of TFPV on UC. Symptoms of DSS-induced colitis in mice manifested as shortened colon length, weight loss, increased DAI index, and histopathological damage of the colon. The experimental results of this study showed that TFPV alleviated the above symptoms of mice induced by DSS. In addition, the results of the detection of inflammatory factors in the serum of DSS-induced UC mice were consistent with those of other models; that is, TFPV down-regulated the expression of pro-inflammatory factors TNF-α, IL-6, and IL-1β, and up-regulated anti-inflammatory factor IL-10 in UC mice. It has been reported that UC occurs when the intestinal mucosa is damaged, and its pathogenesis is related to the intestinal epithelial barrier dysfunction and increased permeability. In the present study, through IHC and WB detection, it was found that DSS significantly reduced the positive expression and protein expression levels of TJ proteins, Claudin, Occludin, and ZO-1, in the colon tissue of mice, while TFPV alleviated this trend. These results suggest that TFPV may play a role in the treatment of UC by restoring the intestinal barrier function through inhibiting the effects of DSS on the expression of inflammatory factors and the thickness of the intestinal mucosal and muscular layers.

Notably, impaired intestinal mucosal barrier, an important feature of ulcerative colitis, is usually associated with targets such as protein kinase B (AKT1), nuclear factor-κB (NF-κB), transcription factor 4 (TCF-4), mitogen-activated protein kinase (MAPK), etc. [54]. Among them, AKT is a subgroup of the Ser/Thr kinase family of AGC proteins, which is a central node for cell signaling downstream of growth factors, cytokines, and other cellular stimuli, and whose role is primarily related to the signaling cascade initiated by phosphatidylinositol 3-kinase (PI3K) activation [55,56]. Activation of the PI3K/AKT signaling pathway induces pro-inflammatory and apoptotic signals mediated by NF-κB, LPS, and TNF-α by enhancing the phosphorylation of IκB (mainly IκBα) and reducing its synthesis, leading to an imbalance in cytokine secretion and amplification of inflammatory cascades [57]. Studies have shown that the PI3K/AKT signaling pathway is closely related to the regulation of tight junction proteins and the maintenance of intestinal epithelial cell barrier function [58,59]. Wang et al. [60] reported that Oleanolic acid 28-O-β-D-glucopyranoside (OAG) inhibited the inflammatory response, increased the expression of tight junction proteins, and enhanced the function of intestinal epithelial barrier in mice with UC, and the inhibition of abnormal activation of the PI3K/AKT signaling pathway was a key mechanism by which OAG could exert the above effects. Li et al. [58], on the other hand, revealed that the effects of hypoxia-preconditioned HF-MSC-derived exosomes (Hy-Exos), such as promoting colonic tight junction protein expression and reducing UC-associated inflammatory injury, were exerted by inhibiting the PI3K/AKT signaling pathway and maintaining mitochondrial function. Consistent with the above studies, the present study showed that AKT is one of the key targets of TFPV acting on UC, and TFPV inhibited the activation of the PI3K/AKT signaling pathway in the colonic tissues of mice caused by DSS. It can be inferred that TFPV may enhance the intestinal barrier function and reduce the expression of pro-inflammatory cytokines by inhibiting PI3K/AKT/NF-κB and IL-17 signaling pathways, thus alleviating the ulcerative colitis in mice induced by DSS.

The intestinal microbiota of patients with UC also tends to show a decrease in beneficial bacteria and an increase in harmful bacteria as the disease progresses [61]. Similarly, dysbiosis of the intestinal flora is common in patients with IBD, which is usually associated with a decrease in bacterial diversity and an imbalance between the various bacterial phyla. It has been reported that short-chain fatty acids are an important energy source of the intestinal epithelial cells, and their deficiency may lead to increased intestinal permeability and trigger inflammation, while microbial dysbiosis is usually one of the reasons for the decrease in short-chain fatty acids in the intestine. Several studies have documented the differences in the composition of gut microbiota between IBD patients and healthy individuals, particularly in terms of microbial diversity and the relative abundance of specific bacterial taxa [62,63]. The intestinal mucosal barrier mainly includes the mechanical barrier, microbial barrier, immune barrier, and chemical barrier [64]. In the present study, 16S rRNA gene sequencing analysis of mouse cecum contents showed that DSS resulted in a significant decrease in Alpha diversity of intestinal microorganisms in mice, which was ameliorated by TFPV. The main communities in the intestine are Firmicutes and Bacteroidota, which account for 90% of the intestinal microbiota [33]. It is widely recognized that the F/B ratio has an important influence on the maintenance of normal intestinal homeostasis. Our results showed that TFPV reduced the increase in the F/B ratio in DSS-induced UC mice, suggesting that TFPV has a regulatory effect on DSS-induced intestinal microbiome dysregulation in mice. In addition, our results also showed that the abundance of Dubosiella in the cecum contents of mice treated with TFPV increased, while the abundance of Enterobacteraceae decreased. Numerous studies have reported that Dubosiella is a beneficial symbiotic bacterium that alleviates the inflammatory response and enhances immunity in mice with colitis [65,66,67]. Specifically, Dubosiella plays a key role in enhancing mucosal barrier integrity, rebalancing the Treg/Th17 response, and maintaining intestinal homeostasis through the production of SCFA (especially propionate) and lysine [68]. Therefore, Dubosiella may be one of the marker microorganisms that TFPV regulates in the gut flora to exert its therapeutic effects on UC. Furthermore, it has been suggested that the abundance of Enterobacteriaceae may be significantly increased in some UC patients, which is consistent with the results of the present experiment [69,70]. Although some Enterobacteriaceae strains are part of the normal gut microbiota, their increased abundance in the intestinal flora of UC patients may be associated with worsening of the disease, inflammatory response, and impaired intestinal barrier [71]. In summary, the alleviating effect of TFPV on DSS-induced UC in mice may be related to its modulation of intestinal flora dysbiosis.

In order to further investigate the metabolic mechanisms by which TFPV addresses DSS-induced UC, an untargeted metabolomics approach utilizing LC-QTOF analysis identified purine metabolism as the most significantly enriched pathway among the differentially expressed metabolites observed between the TFPV and model groups. Purines are essential cellular components that play critical roles in nucleic acid synthesis, DNA replication, and energy metabolism [72]. Notable metabolites within this pathway, including inosine, dGMP, ADP, and uric acid, exhibit interrelated functions in maintaining nucleotide homeostasis. Specifically, inosine serves as an adenine derivative and an intermediate in energy metabolism, dGMP acts as a precursor for DNA synthesis vital for cellular proliferation, and ADP is recognized as a product of ATP degradation involved in energy transfer processes [73].

It is noteworthy that uric acid, the final product of purine metabolism, exhibited a significant reduction in the TFPV group. The dysregulation of purine metabolic enzymes, such as xanthine oxidase, frequently leads to the overproduction of uric acid, which subsequently exacerbates intestinal barrier permeability, a characteristic feature of UC [74]. Metabolomic analyses indicated that TFPV effectively countered the DSS-induced elevations in inosine and ADP levels, as well as the depletion of dGMP. This suggested a dual regulatory function: by normalizing the purine catabolic flux through the modulation of inosine and dGMP levels, TFPV likely reduces the metabolic burden associated with uric acid synthesis. Concurrently, by reducing uric acid levels, TFPV may mitigate intestinal hyperpermeability, thereby restoring the integrity of tight junctions [75], as hyperuricemia has been shown to directly compromise barrier function [76]. This mechanism is further substantiated by the gut–renal axis theory, which posits that dysbiosis of the intestinal microbiota, a significant contributor to UC, disrupts purine catabolism, leading to systemic accumulation of uric acid. The regulation of inosine, ADP, and dGMP by TFPV not only normalizes local purine metabolism but also alleviates uric acid-mediated inflammatory interactions between the gut and the immune system. These findings establish a novel connection between the reprogramming of purine metabolism and the amelioration of UC, highlighting TFPV’s potential as a therapeutic agent for targeting both metabolic and inflammatory pathways.

## 5. Conclusions

In summary, TFPV demonstrates its efficacy in mitigating UC by inhibiting the PI3K/AKT/NF-κB and IL-17 signaling pathways, thereby reducing intestinal inflammation and injury. Moreover, TFPV plays a significant role in maintaining the integrity of the intestinal barrier, regulating the balance of gut microbiota, and lowering uric acid levels. These findings highlight the therapeutic potential of TFPV as a promising agent for the treatment of UC.

## Figures and Tables

**Table 1:** Sequences of primers for RT-qPCR.

Gene	Primer Sequences (5′ to 3′)		Fragment Size (bp)
β-actin	Forward	TTCTAGGCGGACTTGCAGC	128
	Reverse	GCTTCTCAGCAGACAGGAGG	
Claudin-1	Forward	TCTTTCTTATTTCAGGTCTGGCT	91
	Reverse	ACTGGGGTCATGGGGTCATA	
Occludin	Forward	CAGGTGCACCCTCCAGATTG	111
	Reverse	TGGACTTTCAAGAGGCCTGG	
ZO-1	Forward	GAAATACCTGACGGTGCTGC	99
	Reverse	GAGGATGGCGTTACCCACAG	
IL-6	Forward	AAGCTGCAGTCACAGAACGA	136
	Reverse	TGGACGGCATCAATCTCAGG	
IL-10	Forward	CGGCCCAGTGAAGAGTTTCT	98
	Reverse	GGCAACCCAGGTAACCCTTA	
IL-1β	Forward	CCAATTCAGGGACCCTACCC	174
	Reverse	GTTTTGGGTGCAGCACTTCAT	
TNF-α	Forward	GGCCCAAGGACTCAGATCAT	82
	Reverse	CTGTCCCTCGGCTTTGACAT	

**Table 2:** Chemical compositions of TFPV.

NO	Name	Formula	Mass (Da)	[M-H]-	Error (ppm)	Rt (min)
1	DTXSID601174808	C_16_H_23_NO_9_S_2_	437.49	436.07	1.18	0.645
2	N2-(1-Deoxy-I(2)-D-fructopyranos-1-yl)-L-arginine	C_12_H_24_N_4_O_7_	336.34	335.15	1.6	0.662
3	Cotinine N-glucuronide	C_16_H_20_N_2_O_7_	352.34	351.12	2.6	1.062
4	beta-Glucogallin	C_13_H_16_O_10_	332.26	331.06	0.9	1.508
5	Erioflorin methacrylate	C_25_H_22_FNO_3_	403.45	402.15	1	1.913
6	Esculin *	C_15_H_16_O_9_	340.28	339.07	1.18	2.551
7	Pentoxifylline	C_13_H_18_N_4_O_3_	278.31	277.13	0.6	3.36
8	Kaempferin *	C_21_H_20_O_10_	432.38	431.09	0.7	4.201
9	Gallic acid	C_7_H_6_O_5_	170.12	169.01	0.5	5.273
10	Luteolin *	C_15_H_10_O_6_	286.23	285.04	0.71	5.146
11	Bispyribac	C_19_H_18_N_4_O_8_	430.37	429.10	0.79	5.991
12	Procodazole	C_10_H_10_N_2_O_2_	190.2	189.07	0.8	6.285
13	Galangin *	C_15_H_10_O_5_	270.24	269.05	0.8	6.514
14	Querciturone *	C_21_H_18_O_13_	478.36	477.07	0.08	7.635
15	Paucin	C_23_H_32_O_10_	468.49	467.19	0.1	9.163
16	Melanoxetin *	C_15_H_10_O_7_	302.24	301.03	0.18	9.878
17	Tetradecanoic acid	C_14_H_28_O_2_	228.37	227.20	0.3	10.49
18	DTXSID401101613	C_44_H_64_O_16_	848.97	847.41	0.32	11.529
19	Hexadecanamide	C_16_H_33_NO	255.44	254.25	0.5	12.169
20	Furanodiene	C_15_H_20_O	216.32	215.14	0.2	14.889
21	Acrovestone	C_32_H_42_O_8_	554.67	553.28	0.22	15.832
22	10-Gingerol	C_21_H_34_O_4_	350.49	349.28	0.25	16.734
23	L-Ascorbyl 6-Stearate	C_24_H_42_O_7_	442.59	441.28	0.27	18.252
24	Stearyl citrate	C_24_H_46_O_8_	462.62	461.31	0.63	19.631
25	Misoprostol acid	C_21_H_36_O_5_	368.51	367.25	0.45	21.257
26	(6S,7R)-2-azaspiro [5.5] undecan-7-ol	C_10_H_19_NO	169.26	168.14	0.68	21.635
27	Phorbol 12-tiglate 13-decanoate	C_35_H_52_O_8_	600.78	599.36	0.4	22.949
28	Irehine	C_23_H_39_NO	345.56	344.29	0.69	23.265
29	beta-Sitosterol	C_29_H_50_O	414.73	413.38	0.27	25.321
30	1,1′-(1,4-Dihydro-4-nonyl-3,5-pyridinediyl) bis [1-decanone]	C_34_H_61_NO_2_	515.85	514.46	0.31	25.739
31	Uvaricin	C_39_H_68_O_7_	648.95	647.49	1	27.043
32	1,2-Di-O-palmitoyl-3-O-(6-sulfoquinovopyranosyl) glycerol	C_41_H_78_O_12_S	795.12	793.51	0.32	27.13

## Data Availability

Data will be made available upon request.

## Abbreviations

The following abbreviations are used in this manuscript:

ADP	Adenosine Diphosphate
AKT	Protein Kinase B
BP	Biological Processes
CC	Cellular Components
DAI	Disease Activity Index
dGMP	2′-Deoxyguanosine 5′-Monophosphate
DSS	Dextran sulfate sodium
ELISA	Enzyme-Linked Immunosorbent
FC	Fold Change
H&E	Hematoxylin and Eosin
IBD	Inflammatory Bowel Disease
IHC	Immunohistochemical
IL-6	Interleukin-6
IL-10	Interleukin-10
IL-1β	Interleukin-1 Beta
IL-17	Interleukin-17
LPS	Lipopolysaccharide
LC-MS	Liquid Chromatography-Mass Spectrometry
MF	Molecular Functions
MAPK	Mitogen-Activated Protein Kinase
NF-κB	Nuclear Factor-κB
OPLS-DA	Orthogonal Projections to Latent Structures-Discriminant Analysis
PI3K	Phosphatidylinositol 3-Kinase
RT-qPCR	RNA Extraction And Quantitative Real-Time PCR Analysis
TFPV	Total Flavonoids of *Polygonum viviparum* L.
TJ	Tight Junctions
TNF-α	Tumor Necrosis Factor Alpha
UA	Uric Acid
UC	Ulcerative Colitis
VIP	Variable Importance In Projection
